# Novel Zinc(II) Complexes [Zn(atc-Et)_2_] and [Zn(atc-Ph)_2_]: *In Vitro* and *in Vivo* Antiproliferative Studies

**DOI:** 10.3390/ijms17050781

**Published:** 2016-05-21

**Authors:** Erica de O. Lopes, Carolina G. de Oliveira, Patricia B. da Silva, Carlos E. Eismann, Carlos A. Suárez, Amauri A. Menegário, Clarice Q. F. Leite, Victor M. Deflon, Fernando R. Pavan

**Affiliations:** 1 Faculdade de Ciencias Farmaceuticas, UNESP—Univ Estadual Paulista, Campus Araraquara, Araraquara, São Paulo 14800-903, Brasil; ericaoliveir@bol.com.br (E.d.O.L.); patrbent@yahoo.com.br (P.B.d.S.); claricequeico@gmail.com (C.Q.F.L.); 2 Instituto de Química de São Carlos, USP—Univ de São Paulo, São Carlos, São Paulo 13560-970, Brasil; carolsatrini@gmail.com (C.G.d.O.); deflon@iqsc.usp.br (V.M.D.); 3 Centro de Estudos Ambientais, UNESP—Univ Estadual Paulista, Campus Rio Claro, Rio Claro, São Paulo 13506-900, Brasil; kadueismann@gmail.com (C.E.E.); suarezcarlos@yahoo.com (C.A.S.); amenega@rc.unesp.br (A.A.M.)

**Keywords:** zinc(II) complexes, cancer, antiproliferative activity, *Artemia salina* L., acute toxicity, oral bioavailability

## Abstract

Cisplatin and its derivatives are the main metallodrugs used in cancer therapy. However, low selectivity, toxicity and drug resistance are associated with their use. The zinc(II) (Zn^II^) thiosemicarbazone complexes [Zn(atc-Et)_2_] (**1**) and [Zn(atc-Ph)_2_] (**2**) (atc-R: monovalent anion of 2-acetylpyridine N4-R-thiosemicarbazone) were synthesized and fully characterized in the solid state and in solution via elemental analysis, Fourier transform infrared (FTIR), ultraviolet-visible (UV-Vis) and proton nuclear magnetic resonance (^1^H NMR) spectroscopy, conductometry and single-crystal X-ray diffraction. The cytotoxicity of these complexes was evaluated in the HepG2, HeLa, MDA-MB-231, K-562, DU 145 and MRC-5 cancer cell lines. The strongest antiproliferative results were observed in MDA-MB-231 and HepG2 cells, in which these complexes displayed significant selective toxicity (3.1 and 3.6, respectively) compared with their effects on normal MRC-5 cells. *In vivo* studies were performed using an alternative model (*Artemia salina* L.) to assure the safety of these complexes, and the results were confirmed using a conventional model (BALB/c mice). Finally, tests of oral bioavailability showed maximum plasma concentrations of 3029.50 µg/L and 1191.95 µg/L for complexes **1** and **2**, respectively. According to all obtained results, both compounds could be considered as prospective antiproliferative agents that warrant further research.

## 1. Introduction

Cancer consists of a complex group of diseases in which uncontrolled cell growth invades tissues and organs and potentially spreads to other regions of the body (metastasis). Mutated cells divide rapidly and tend to behave aggressively, causing the formation of tumors or malignancies [1]. Today, cancer is responsible for one out of every eight deaths worldwide—more than human immunodeficiency virus (HIV)/acquired immune deficiency syndrome (AIDS), tuberculosis and malaria combined. Therefore, cancer is clearly a public health problem. The incidence of cancer is growing at an alarming rate, and simply due to the growth and aging of the world population, approximately 21.7 million new cases and 13.0 million deaths are expected by 2030 [2]. The modes of cancer treatment include surgery, chemotherapy, radiotherapy, transplantation, targeted therapy, immune therapy, and photodynamic therapy. Chemotherapy is an important cancer treatment option. However, its main drawback is non-selective toxicity, as chemotherapeutic agents act on both tumor cells (the targets) and normal cells [3].

The development of cisplatin represents one of the most significant advances in the treatment of various cancers. Since its approval in 1978 by the U.S. Food and Drug Administration (FDA), it has been widely used to treat a variety of solid and hematological tumors. However, there are problems associated with its use related to its low therapeutic index and high potential toxicity to the kidneys (nephrotoxicity) and the gastrointestinal tract [4,5]. Due to the frequent use of cisplatin alone or in combination with other chemotherapy drugs, drug resistance is observed in numerous types of cancer, such as ovarian cancer, lung cancer, pancreatic cancer and nasopharyngeal carcinoma [6,7,8,9]. Thus, several drug resistance mechanisms, including drug efflux, inhibition of apoptosis, and increased DNA repair, have been studied and described [10].

The study of inorganic chemistry in recent years has been demonstrated to be effective in the development of new therapeutic agents. Drugs based on metals are increasing in importance in therapies for cancer and other diseases [11,12]. It is known that certain metals such as zinc (Zn), a trace element in the human body, participate in various reactions in biological systems, in addition to their presence in metalloenzymes fundamental for our body [13]. Studies have noted that the activity of bio-metals, such as Zn, is modified after the formation of coordination compounds, and the thermodynamic and kinetic properties of these metals can also be modified to regulate their biological activities. These properties include permeability, lipophilicity and formation constants, which can be critical for transition metals in reaching target sites [14]. Previously studied Zn complexes showed antiproliferative activity on different tumor cell lines that was greater than that of their free ligands [15].

Within this context, our group has studied the effect of metal coordination on bioactivity. In the present study, we synthesized and characterized new Zn^II^ complexes, [Zn(atc-Et)_2_] (**1**) and [Zn(atc-Ph)_2_] (**2**), and evaluated their *in vitro* antiproliferative activity, their toxicological safety using an alternative model (*Artemia salina* L.) as well as a BALB/c mouse model and, finally, their *in vivo* oral bioavailability using a rapid screening method.

## 2. Results and Discussion

### 2.1. Chemistry

**Scheme 1:**
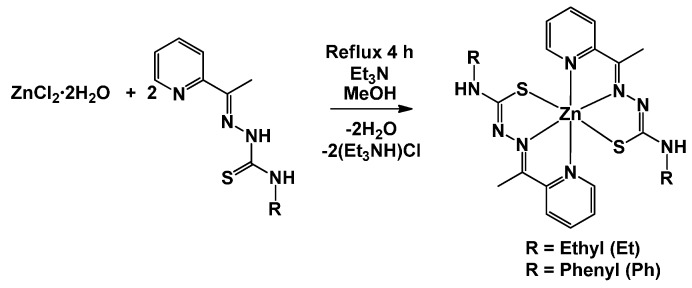
Synthesis of the complexes.

Reacting Hatc-Et or Hatc-Ph with ZnCl_2_·2H_2_O in the presence of Et_3_N under reflux in MeOH produced pure yellow precipitates of the Zn complexes at reasonable yields (Scheme 1). Complex **1** was very soluble in CH_2_Cl_2_ but was less soluble in methanol or ethanol. Alternatively, complex **2** was reasonably soluble only in dimethyl sulfoxide (DMSO). Elemental analyses and molar conductivity measurements produced results in accordance with the formation of neutral complexes of the type [Zn(atc-R)_2_].

**Figure 1:**
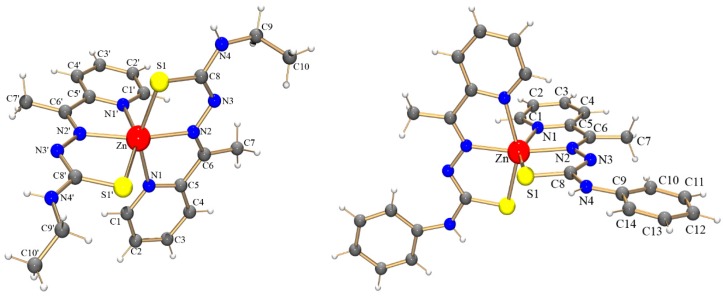
Molecular structure determined for [Zn(atc-Et)_2_] (**1**) (**left**) and [Zn(atc-Ph)_2_] (**2**) (**right**) with atom labels.

The Fourier transform infrared (FTIR) and proton nuclear magnetic resonance (^1^H NMR) spectra of these complexes were consistent with the *N*,*N*,*S*-coordination of the atc-R^1−^ ligands to the Zn^II^ metal center. Single-crystal X-ray diffraction was performed to determine the structures of the complexes (Figure 1). Table 1 summarizes selected bond lengths and angles. The thiosemicarbazone ligands coordinate to Zn^II^ via their pyridine and azomethine nitrogen atoms as well as their sulfur atom, forming two four-membered chelated rings with a metal center. In both complexes, the tridentate ligands are nearly planar and perpendicular to each other. The coordination spheres of both complexes are best described as possessing a distorted octahedral geometry.

The Zn-N bond lengths are similar but slightly shorter when involving the azomethine nitrogen atom. The Zn-S bonds for complexes **1** and **2**, with lengths of approximately 2.45 Å, are very similar to those observed in similar complexes reported in the literature [16]. The bond lengths involving the donor atoms and the Zn^II^ metal center in the complex [Zn(atc-Et)_2_] are slightly shorter than those in the analogous Mn^II^ complex [Mn(atc-Et)_2_] [17]. This result is in agreement with the smaller ionic radius of Zn^II^ than that of Mn^II^. In the Co^III^ cationic complex [Co(atc-Et)_2_]^+^, the bond lengths surrounding the metal center remain shorter than those in similar complexes containing Mn^II^ and Zn^II^, as expected [18].

### 2.2. Cytotoxic Activity toward MRC-5, HepG2, HeLa, MDA-MB-231, K-562 and DU 145 Cell Lines

The search for new molecules that exhibit selectivity for a tumor cell but not a normal cell is a major challenge in anticancer drug research [19]. Therefore, we are seeking new complexes with selectivity indices of greater than three, indicating that they are cytotoxic to tumor cells at a concentration at least three-fold lower than the concentration in which they are cytotoxic to normal cells [20]. Concentration inhibiting cellular proliferation by 50% (IC_50_) values are presented in Table 2. For comparison purposes, the cytotoxicity of cisplatin as a reference antitumor drug was also evaluated under the same conditions.

The free ligand Hatc-Et and the precursor salt ZnCl_2_·6H_2_O was cytotoxic to normal MRC-5 cells and showed no effects on all tumor cells tested; therefore, these compounds were considered to be inactive on tumor cells. Alternatively, Hatc-Ph displayed activity on the tumor cell lines as well as cytotoxicity to MRC-5 cells, resulting in low selectivity. In two cases, the coordination of the ligand to Zn led to the formation of complexes with higher antiproliferative activities. Complex **1** showed selective cytotoxicity to the MDA-MB-231 cell line with a selectivity index (SI) of 3.1, and complex **2** showed an SI of 3.6 in the HepG2 cell line. Both complexes were demonstrated to be more active and selective than cisplatin in all cell lines tested.

Importantly, DMSO (solvent control) alone showed no cytotoxic activity toward the MRC-5, HepG2, HeLa, MDA-MB-231, K-562 and DU 145 cell lines.

There is no report available in the literature regarding the SI of the antitumor activities of Zn^II^ complexes, and this lack of data prevented us from comparing the results obtained in this study with previous findings.

### 2.3. Toxicity in Artemia salina L.

Complexes **1** and **2** showed 3.3- and 5.7-fold lower toxicity in the microcrustacean *Artemia salina* L., respectively, relative to cisplatin. The 50% lethal concentration (LC_50_) values, defined as the concentration of a substance causing lethality to 50% of the organisms exposed, are presented in Table 3.

There are a few reports in the literature using *Artemia salina* L. to determine the toxicity of novel substances, and most of these studies were conducted using plant extracts [21,22]. However, with the increasing need to use alternative models, new toxicity studies of organic and inorganic molecules using microcrustaceans as model organisms are emerging [23,24].

The Zn^II^ complexes investigated in this study were less toxic to the microcrustacean than the perchlorate compound of bis(isoniazid) Zn^II^ hexahydrate (LC_50_ = 268 µM), as reported in the literature by Freitas *et al.* (2011) [25].

Copper(II) (Cu^II^) complexes with 2-pyridineformamide-derived thiosemicarbazones studied by Ferraz *et al.* (2009) showed LC_50_ values ranging from 1.2 to 7.2 µM against *Artemia* *salina* L.; those values smaller than the LC_50_ values observed for the present Zn^II^ complexes [23]. A comparison between the data on cytotoxicity to cell lines and toxicity in *Artemia* *salina* L. in the present study indicated that complexes **1** and **2** showed selectivity toward the MDA-MB-231 and HepG2 cell lines, respectively, but that neither complex was toxic to the microcrustacean. DMSO, used as the solvent, was not toxic to *Artemia* *salina* L.

### 2.4. Acute Toxicity in Vivo

The samples were used at a dose of 1000 mg/kg due to the low solubility of the complexes in sunflower oil, which further hindered the performance of the gavage technique in the animals. The Globally Harmonized System of Classification and Labelling of Chemicals (GSH) classifies oral toxic agents into five categories, and the fourth category includes the substances that display median lethal dose (LD_50_) >300 mg/kg ≤2000 mg/kg. Therefore, the dose used in our experiments is within the range recommended by the Organization for Economic Cooperation Development (OECD) (2001) [26].

**Figure 2:**
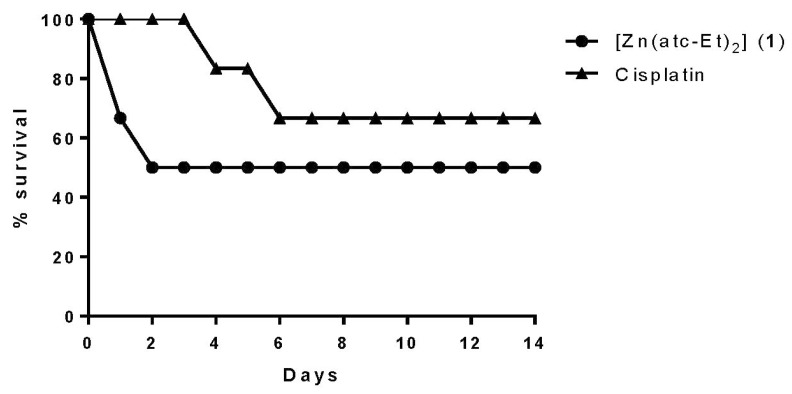
Percentage survival of BALB/c mice over 14 days of drug treatment at the concentration of 500 mg/kg body weight.

**Figure 3:**
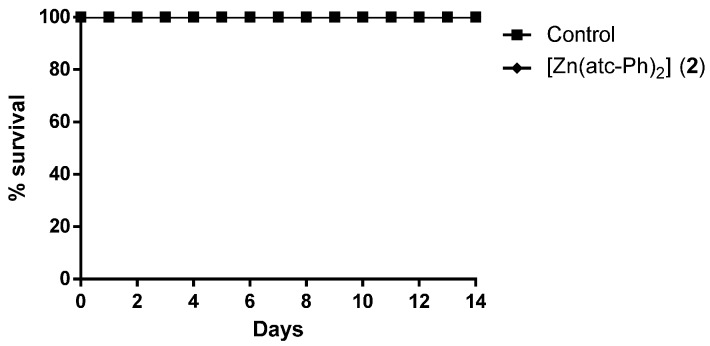
Percent survival of BALB/c mice over 14 days of drug treatment at the concentration of 1000 mg/kg body weight.

Complex **1** at a dose of 1000 mg/kg body weight caused mortality in all BALB/c mice during the experiment, so it was necessary to administer a dose of 500 mg/kg body weight via gavage to determine its LD_50_ (Figure 2). The solvent control and complex **2** at the concentration of 1000 mg/kg body weight showed no mortality, as shown in Figure 3. Cisplatin led to the death of two animals when administered at the dose of 500 mg/kg body weight. The effects of cisplatin at higher concentrations are reported in the literature [27].

The animals were monitored over 14 days of drug treatment, and behavioral parameters were evaluated (Hippocratic screening). However, no behavioral changes were observed.

According to the data obtained in this study concerning the toxicity of these complexes to *Artemia salina* L. and BALB/c mice, the results are in agreement. Specifically, complex **2** was less toxic than complex **1** in both the alternative and *in vivo* models.

Organ weight has long been accepted as a sensitive indicator of chemically induced changes to organs. In toxicological experiments, comparison of organ weights between treated and untreated groups of animals have conventionally been used to evaluate the toxic effect of a treatment [28,29].

**Figure 4:**
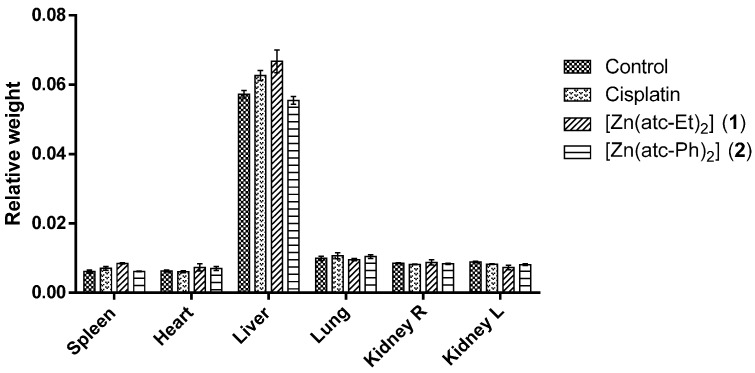
Relative weight (mean ratio of organ weight to body weight of BALB/c mice) and standard deviation (SD). R: right ; L: left.

Macroscopic analysis of the organs showed no morphological changes and revealed no statistically significant difference in the mean ratio of organ weight to body weight among the animals treated with either of the Zn^II^ complexes or cisplatin compared to the control animals (Figure 4).

### 2.5. Quantification of the Enzymatic Activity Levels of Aspartate Aminotransferase (AST) and Alanine Aminotransferase (ALT) in the Serum of BALB/c Mice

Quantification of enzymatic activity levels of AST and ALT is important in the diagnosis of heart and liver damage caused by heart attack, drug toxicity, or infection. AST is present in other tissues such as heart, skeletal muscle, kidney, brain, and pancreatic tissues. Therefore, AST is much more widely expressed than ALT, which is primarily expressed in the liver. Therefore, upon liver damage, both enzymes are released into serum/plasma. In contrast, if the lesion is purely hepatic, the serum ALT level increases slightly more than the serum AST level [30].

**Figure 5:**
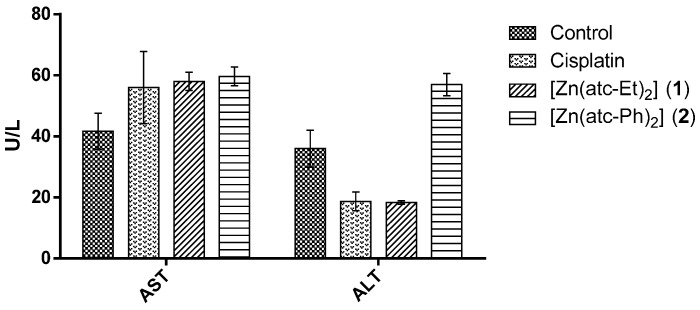
Quantification of the serum aspartate aminotransferase (AST) and alanine aminotransferase (ALT) activity levels in BALB/c mice. U/L: units per liter.

Regarding the enzymatic activity levels (of AST and ALT) in serum from mice, no significant difference was observed between the groups treated with either of the Zn^II^ complexes or with cisplatin and the control group, as shown in Figure 5 and as demonstrated in another study [31]. This result confirms that no change in liver toxicity occurred in BALB/c mice.

As shown in Figure 5, the levels of ALT in the samples from mice treated with cisplatin or complex **1** were the half of the control levels. However, analysis of variance (ANOVA) and Dunnett’s test did not indicate that these differences were significant (*p* > 0.05).

### 2.6. Oral Bioavailability in Vivo

The *in vivo* test of oral bioavailability consists of a rapid screen that reveals whether an administered complex or the final product was absorbed into the bloodstream. The calibration curves used for quantitative evaluation of plasma samples showed an R^2^ correlation coefficient of 0.999, and the detection limit (DL) for Zn metal was 480 µg/L. For cisplatin (platinum (Pt) of atomic mass 195 u), the DL was 0.0003954 µg/L, and the quantification limit (QL) was 0.001318 µg/L.

**Figure 6:**
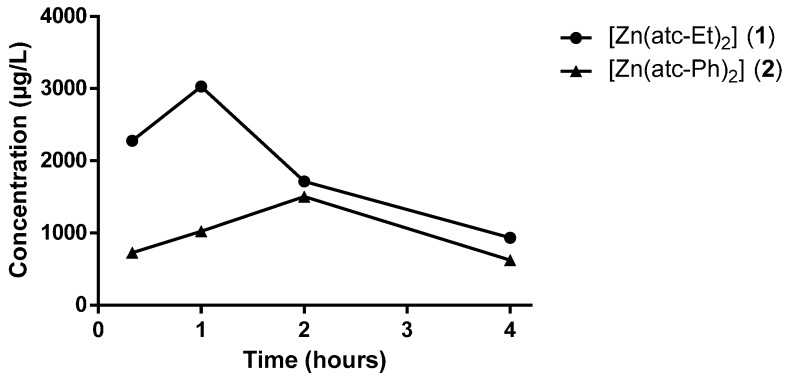
Time-concentration profiles of Zn^II^ complexes in plasma after a single administration via oral gavage at a dose of 300 mg/Kg body weight.

Figure 6 shows the time-concentration profiles for complexes **1** and **2** in plasma. The values presented in the figure were calculated relative to the control sample because the body itself contains a baseline concentration of Zn.

The plasma concentrations of complex **1** at 20 min, 1 h, 2 h and 4 h after administration were 2278.50 µg/L, 3029.50 µg/L, 1715.50 µg/L and 935.55 µg/L, respectively. The concentration of the complex at 20 min was the peak concentration observed (Cmax). For complex **2**, the plasma concentrations at the same sampling time points were 727.83 µg/L, 1023.55 µg/L, 1191.95 µg/L and 624.70 µg/L, respectively. For this complex, Cmax in plasma was detected at 2 h, after which the plasma concentration of complex **2** decayed.

The time-concentration profiles of both complexes are in agreement with the findings of a previous study [32]. The differences in the profiles of Zn^II^ complexes **1** and **2** observed in this study are probably due to their structural differences regarding the composition of their ligands.

**Figure 7:**
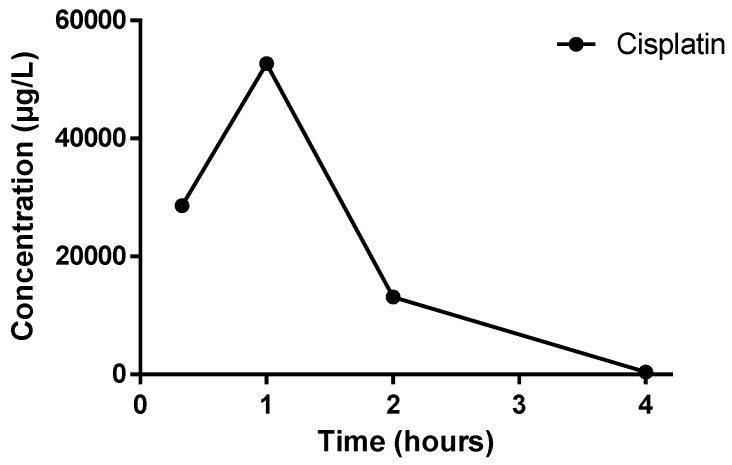
Time-concentration profile of cisplatin in plasma after a single administration via oral gavage at a dose of 300 mg/Kg body weight.

The platinum (Pt) concentration was quantified to determine the time of Cmax for the orally bioavailable drug cisplatin (Figure 7); Cmax of platinum ((Pt) of atomic mass 195 u)was determined to be 1 h after cisplatin administration.

The complex profiles show that **1** and cisplatin display similar time points of Cmax occurrence (Tmax, time of maximum compound concentration), both of which were approximately 1 h. In contrast, **2** displayed a Tmax of 2 h.

## 3. Experimental

### 3.1. Inorganic Complexes

2-Acetylpyridine, 4-ethyl-3-thiosemicarbazide, 4-phenyl-3-thiosemicarbazide, ZnCl_2_·2H_2_O and solvents were obtained commercially and used without further purification. The ligands Hatc-Et and Hatc-Ph were prepared as reported [17,18]. FTIR spectra between 400 and 4000 cm^−1^ were measured via the Potassium Bromide (KBr) pellet method using a Shimadzu IR Prestige-21 spectrophotometer (Shimadzu^®^, Tsukinowa, Otsu, Japan). Elemental analyses were performed using a Perkin-Elmer CHN 2400 elemental analyzer (Perkin Elmer^®^, Waltham, MA, USA). The conductivities of the complexes were measured in CH_2_Cl_2_ solution using an Orion Star Series conductometer (Thermo Scientific^®^, San Diego, CA, USA). Ultraviolet-visible (UV-Vis) spectra of solutions diluted in DMSO were measured using a Shimadzu UV-1800 spectrophotometer (Shimadzu^®^, Tsukinowa, Otsu, Japan). Proton nuclear magnetic resonance (^1^H NMR) spectra were acquired using an Agilent 400/54 Premium Shielded 9.4 T NMR spectrometer (Agilent^®^, Santa Clara, CA, USA), set to 399.8 MHz for ^1^H. The NMR spectra were internally referenced to the spectrum of Tetramethylsilane (TMS). Yellow crystals were produced via slow evaporation of the mother solution of the two Zn^II^ complexes at room temperature. Data collection was performed at 296 K by applying Mo-Kα radiation (λ = 71.073 pm) using a Bruker Kappa APEX II Duo diffractometer (Bruker^®^, Karlsruhe, Germany). The multi-scan method was applied for absorption correction. The structures were solved with SHELXS97 software (Gottingen, Germany) [33] using direct methods, and all non-hydrogen atoms were refined with anisotropic displacement parameters in SHELXL97. The hydrogen atoms were calculated at idealized positions using the riding model option of SHELXL97 [33]. The asymmetric unit in the crystal structure of complex **2** contains only half of the entire molecule. Table 4 presents more detailed information about the structure determinations. The structural data were deposited at The Cambridge Crystallographic Data Centre (CCDC) as entries CCDC 1436883 (1) and CCDC1436884 (2), and these data can be obtained free of charge from The Cambridge Crystallographic Data Centre.

### 3.2. Synthesis of the Complexes

The Zn^II^ complexes were synthesized by adding one equivalent (0.25 mmol) of ZnCl_2_·2H_2_O to a solution containing two equivalents (0.50 mmol) of the ligand Hatc-R in 15 mL of MeOH supplemented with 3 drops of Et_3_N. The resulting solutions were stirred for 4 h under reflux. Subsequently, the yellow precipitate that formed was filtered off, washed with cold methanol and hexane and dried under vacuum. Via slow concentration of the mother solutions, yellow crystals suitable for X-ray diffraction were obtained for both complexes.

[Zn(atc-Et)_2_] (**1**): Color: Yellow. Yield: 0.078 g (61%). Analysis: found: C, 46.74%; H, 5.15%; and N, 23.05%; calculated for C_20_H_26_N_8_S_2_Zn (507.98 g/mol): C, 47.29%; H, 5.16%; and N, 22.06%. IR (ν_max_/cm^−1^): 3,216 ν (N−H), 1,594, 1,555 ν (C=N) + ν (C=C), 1,074 ν (N–N), and 781 ν (C−S). ^1^H NMR (DMSO, 399.8 MHz): δ 1.14 (t, *J* = 8 Hz, CH_3_CH_2_, 6H), 2.45 (s, CH_3_C=N, 6H), 3.43 (q, *J* = 8 Hz, CH_2_CH_3_, 4H), 7.44 (s, NHCH_2_, 2H), 7.58 (t, *J* = 6 Hz, Py, 2H), 7.83 (d, *J* = 8 Hz, Py, 2H), 8.09 (t, *J* = 8 Hz, Py, 2H), and 8.65 (d, *J* = 8 Hz, Py, 2H). UV-Vis peaks at 2.16 × 10^−5^ M in DMSO solution [λ_max_ (ε, M^−1^·cm^−1^)]: 398.00 nm (3611) and 310.00 nm (3141). Molar conductivity (at 1 × 10^−3^ M in dichloromethane): 0.10 μS·cm^−1^.

[Zn(atc-Ph)_2_] (**2**): Color: Yellow. Yield: 0.077 g (50%). Analysis: found: C, 54.73%; H, 4.33%; and N, 18.23%; calculated for C_28_H_26_N_8_S_2_Zn (604.07 g/mol): C, 55.67%; H, 4.34%; and N, 18.55%. IR (ν_max_/cm^−1^): 3,255 ν (N−H), 1,596, 1,548 ν (C=N) + ν (C=C), 1,084 ν (N–N), and 781 ν (C−S). ^1^H NMR (DMSO, 399.8 MHz): δ 2.71 (s, CH_3_C=N, 6H), 6.94 (t, *J* = 8Hz, Ph, 2H), 7.28 (t, *J* = 8Hz, Ph, 4H), 7.39 (t, *J* = 8 Hz, Py, 2H), 7.87–7.96 (m, Py + Ph, 10H), and 9.29 (s, NHPh, 2H). UV-Vis peak at 2.15 × 10^−5^ M in DMSO solution [λ_max_ (ε, M^−1^·cm^−1^)]: 396.50 nm (3,265). Molar conductivity (at 1 × 10^−3^ M in dichloromethane): 0.13 μS·cm^−1^.

### 3.3. Cell Culture

All adherent cell lines, including healthy lung fibroblast (MRC-5), hepatocellular carcinoma (HepG-2), cervix adenocarcinoma (HeLa), breast adenocarcinoma (MDA-MB-231), prostate carcinoma (DU 145) and suspended chronic myelogenous leukemia (K-562) cells, were cultured in a humidified atmosphere containing 5% CO_2_ at 37 °C. The cultures were maintained in Dulbecco's Modified Eagle Medium (DMEM) or Roswell Park Memorial Institute (RPMI) 1640 medium (Vitrocell^®^, Campinas, SP, Brazil) supplemented with 10% fetal bovine serum (FBS), 50 mg/L gentamicin sulfate, and 2 mg/L amphotericin B.

### 3.4. Cytotoxic Analysis (IC_50_) of Adherent Cell Lines

In these experiments, cells were collected in a solution of trypsin/ethylenediaminetetraacetic acid (EDTA) (Vitrocell^®^) and centrifuged (252× *g* for 5 min). The number of cells was counted using a Neubauer chamber (Celeromics, Valencia, Spain) after staining non-viable cells with 0.4% trypan blue solution (Sigma-Aldrich^®^) via the cell exclusion assay. Then, the cell concentration was adjusted to 7.5 × 10^4^ cells/mL in DMEM for tumor cells and MRC-5 cells. Next, a 200 μL suspension was deposited into each well of a 96-well microplate to a cell density of 1.5 × 10^4^ cells/well. The cells were incubated at 37 °C in an atmosphere of 5% CO_2_ for 24 h to allow the cells to attach to the plate [34]. The coordination compounds were solubilized in DMSO to an initial concentration of 10,000 µg/mL. Test solutions of the complexes were prepared to obtain concentrations from 500 to 1.95 μg/mL. The diluted solutions were added to the cells after changing the medium to remove any non-adherent cells, and the cultures were incubated for an additional 24 h. The cytotoxicity of the complexes was determined after incubating the cells in 30 μL of resazurin for approximately 2 h. The measurement was performed using a Synergy H1 microplate reader (BioTek^®^, Winooski, VT, USA) with excitation and emission filters at wavelengths of 530 and 590 nm, respectively. The assays were performed in three independent experiments.

As the reference and the control, 10,000 µg/mL cisplatin (Sigma-Aldrich^®^) and 5% DMSO, respectively, were used in all cytotoxicity assays.

### 3.5. Cytotoxic Analysis (IC_50_) of Suspended Cells

In these experiments, cells were centrifuged (252× *g* for 5 min), and the number of cells was counted using a Neubauer chamber after staining non-viable cells with 0.4% trypan blue solution (Sigma-Aldrich^®^) via the cell exclusion assay. Then, the cell concentration was adjusted to 2 × 10^5^ cells/mL in RPMI 1640 medium. Next, 100 μL of the suspension was deposited into each well of a 96-well microplate to a final cell density of 2 × 10^6^ cells/well [35]. The complexes were dissolved in DMSO to an initial concentration of 10,000 μg/mL. Test solutions of the complexes were prepared to obtain concentrations from 500 to 1.95 μg/mL. The diluted solutions were added to the cells, and the cultures were incubated for 24 h. The cytotoxicity of the complexes was determined after incubating the cells in 30 μL of resazurin for approximately 5 h. The measurement was performed using a Synergy H1 microplate reader (BioTek^®^, Winooski, VT, USA) with excitation and emission filters at wavelengths of 530 and 590 nm, respectively. The assays were performed in three independent experiments.

In all cytotoxicity assays, as the reference and the control, cisplatin (Sigma-Aldrich^®^) at a concentration of 10,000 µg/mL and 5% DMSO (Sigma-Aldrich^®^), respectively, were used.

### 3.6. SI Measurement

The SI of the complexes was calculated as the ratio of the IC_50_ for healthy MRC-5 cells to the IC_50_ for tumor cells. A greater IC_50_ value indicates that the compound is more active against tumor cells relative to normal host cells. Substances with SI values >3 are considered promising [20].

### 3.7. Artemia salina L. Toxicity Assay

To determine toxicity of the Zn^II^ complexes to brine shrimp, 25 mg of egg (Natal, RN, Brazil) was incubated in a beaker (2000 mL) containing artificial salt water at a temperature ranging from 20 to 30 °C. The artificial salt water consisted of 23 g NaCl, 11 g MgCl_2_·6H_2_O, 4 g Na_2_SO_4_, 1.3 g CaCl_2_·H_2_O, and 0.7 g KCl in 1000 mL distilled water. The pH was adjusted to 9.0 using 5N NaOH to avoid risk of death to the *Artemia* larvae (nauplii) due to a low pH during incubation.

After 24 hours, 0.6 g of *Saccharomyces cerevisiae* was added to the beaker per liter of salt water to feed the nauplii; after the eggs were incubated for 48 h, the nauplii were extracted for the experiments.

A 120 µL suspension of 10-15 nauplii was added to each well of a 96-well microplate, including the control wells. The compounds were solubilized in DMSO at 15,000 µg/mL and serially diluted in a separate 96-well microplate. The tested concentrations ranged from 1500 to 5.85 µg/mL. After incubation for 24 h, the plates were examined under a Lupa binocular microscope (X3.0), and the numbers of live nauplii in each well were counted to determine the LC_50_ [21]. The assays were performed in three independent experiments.

In all toxicity assays, 15,000 µg/mL cisplatin (Sigma-Aldrich^®^) was used as the reference drug, and 2% DMSO (Sigma-Aldrich^®^) was used as a control at.

### 3.8. In Vivo Oral Bioavailability Tests

Female 4–8-week old BALB/c mice weighing 15–20 g were purchased from the central animal facilities of Campinas State University (UNICAMP, Campinas, SP, Brazil). The mice were maintained in polycarbonate cages at 23 ± 2 °C, 56% ± 2% humidity at a 12 h light/dark cycle under specific pathogen-free conditions (in a positive-pressure cabinet), and the animals were provided with food and water *ad libitum*. The *in vivo* oral bioavailability of the complexes was determined according to a previously suggested method [36]. The animals (*n* = 2 animals/complex) received a dose of 300 mg/Kg body weight via oral gavage. At 20 min, 1 h, 2 h, and 4 h after drug administration, blood was drawn from two animals via the retro-orbital veins, draining the submandibular vein and the face of the mice to the point at which the origins of the jugular vein join so that drops of blood exude from the point of penetration [37]. The plasma was separated and stored at −70 °C for quantification.

### 3.9. Specimen Preparation for Inductively Coupled Plasma Optical Emission Spectrometry (ICP-OES)

The metal ion (Zn) concentration was quantified via ICP-OES using a Thermo Scientific Model 6300 system (Thermo Scientific^®^, San Diego, CA, USA). Samples (50 µL) were diluted 1:100 with a solution containing 0.01% (*v/v*) Triton X-100 and 0.5% (*v*/*v*) nitric acid [38]. During the experiment, the recovery validation test was performed, and this test produced a result of 94%–104% recovery.

### 3.10. Specimen Preparation for Inductively Coupled Plasma Mass Spectrometry (ICP-MS)

The concentration of the drug cisplatin was quantified via ICP-MS using a Thermo Scientific XSeries II system (Thermo Scientific^®^, San Diego, CA, USA). For digestion, 50 µL of plasma sample and 5950 µL of double-distilled 20% (*v*/*v*) nitric acid were used. The samples were digested in a microwave (Berghof speedwave D 72800, Goshen, NY, USA), reaching a temperature of 170 °C. During the experiment, the recovery validation test was performed, and this test produced a result of 95%–105% recovery.

### 3.11. In Vivo Acute Toxicity Assay

Female 16–20-week old BALB/c mice weighing 24–26 g were used after utilizing these animals for the bioavailability experiments. As described by Gruppo *et al.* (2006), the surviving animals were reused to reduce the number of animals required for research. The mice were maintained in polycarbonate cages at 23 ± 2 °C, 56% ± 2% humidity at a 12-h light/dark cycle under specific-pathogen-free conditions (in a positive-pressure cabinet), and the animals were provided with food and water *ad libitum* [36].

The safety profile of the complexes to BALB/c mice was evaluated via acute toxicity assays according to protocols established by the OECD using the classical method to measure the acute toxicity (LD_50_) of a single oral dose (administered via gavage) of the complexes [26].

Each complex was suspended in a sunflower oil vehicle, and the vehicle was used as a control (control group). The complexes were administered as a single dose at 1000 mg/kg body weight via gavage (*n* = 6 animals/complex). When treatment with any complex resulted in greater than 50% mortality, doses of 500 and 300 mg/kg body weight were administered. The animals were monitored for 14 days, and behavioral parameters (Hippocratic screening) were evaluated.

At the end of the experiment, the animals were weighed, and blood was collected via the submandibular vein. Subsequently, the animals were euthanized in CO_2_ chambers, and their organs (heart, lungs, right and left kidneys, spleen and liver) were surgically removed, weighed and macroscopically analyzed. Statistical analysis was performed using GraphPad Prism Version 1.5 software (La Jolla, CA, USA) via ANOVA and Dunnett’s test, and *p* < 0.05 was established as the significance level.

### 3.12. Quantification of the Serum AST and ALT Enzymatic Activity Levels in BALB/c Mice

To analyze whether there were any biochemical changes in the liver of the animals, we quantified the serum AST and ALT enzymatic activity levels in mice after administration of the complexes via gavage before sacrificing, as described above. A total of 500 µL of blood was collected via the submandibular vein. The blood samples were placed in collection tubes, centrifuged at 1097.6× *g* for 15 min to separate the serum, and stored at −70 °C. The samples were analyzed on the day of collection.

Enzymatic activity measurements were performed using biochemical diagnostic kits from Labtest S.A. (Labtest S.A., Lagoa Santa, MG, Brazil).

Statistical analysis was performed using GraphPad Prism Version 1.5 software via ANOVA and Dunnett’s test, and *p* < 0.05 was established as the significance level.

## 4. Conclusions

In conclusion, the results indicate that the Zn^II^ complexes [Zn(atc-Et)_2_] and [Zn(atc-Ph)_2_] might be a promising source of new metal-based antitumor agents. Based on the investigated toxicity parameters, the complexes have been demonstrated to be safe to BALB/c mice when orally administered at the doses tested. In addition, we determined the times of Cmax of the Zn^II^ complexes after administration via oral gavage: one hour for complex **1** and two hours for complex **2**.

## Figures and Tables

**Table 1:** Selected bond lengths (Å) and angles (°) for [Zn(atc-Et)_2_] (**1**) and [Zn(atc-Ph)_2_] (**2**).

	1	2
**Bond Lengths**		
Zn(1)-N(2)/Zn(1)-N(2’)	2.1586(18)/2.1527(17)	2.1549(17)
Zn(1)-N(1)/Zn(1)-N(1’)	2.203(2)/2.2482(18)	2.2139(18)
Zn(1)-S(1)/Zn(1)-S(1’)	2.4539(10)/2.4464(9)	2.4452(6)
S(1)-C(8)/S(1’)-C(8’)	1.739(2)/1.719(2)	1.729(2)
N(3)-C(8)/N(3’)-C(8’)	1.321(3)/1.338(3)	1.323(3)
**Angles**		
N(2’)-Zn(1)-N(2)	161.22(5)	169.60(9)
N(2’)-Zn(1)-N(1)	104.79(7)	98.88(6)
N(2)-Zn(1)-N(1)	73.95(7)	73.62(7)
N(2’)-Zn(1)-N(1’)	73.58(7)	73.62(7)
N(2)-Zn(1)-N(1’)	87.64(7)	98.88(6)
N(1)-Zn(1)-N(1’)	87.97(6)	90.55(9)
N(2’)-Zn(1)-S(1’)	79.18(6)	79.08(5)
N(1)-Zn(1)-S(1)	151.47(5)	152.54(5)
N(1’)-Zn(1)-S(1’)	150.67(5)	152.54(5)
C(8)-S(1)-Zn(1)	96.13(8)	95.93(7)
C(1)-N(1)-C(5)	118.95(19)	118.58(19)

**Table 2:** Cytotoxicity (IC_50_) of ZnCl_2_·6H_2_O, free ligands, complexes **1** and **2** and cisplatin against tumor cell lines and normal (MRC-5) cells.

Complexes	IC_50_ (µM) ± SD										
	MRC-5 ^1^	HepG2 ^2^	SI *	HeLa ^2^	SI *	MDA-MB-231 ^2^	SI *	K-562 ^2^	SI *	DU 145 ^2^	SI *
ZnCl_2_·6H_2_O	239.9 ± 16.8	698.4 ± 10.9	0.3	864.5 ± 14.0	0.3	475.4 ± 64.3	0.5	1088.7 ± 73.8	0.2	382.9 ± 7.8	0.6
Hatc-Et	9.0 ± 0.0	552.8 ± 45.0	0.0	746.0 ± 23.2	0.0	903.5 ± 65.3	0.0	420.3 ± 3.7	0.0	531.5 ± 12.6	0.0
Hatc-Ph	7.4 ± 0.0	36.3 ± 1.5	0.2	25.0 ± 0.2	0.3	24.3 ± 5.0	0.3	11.1 ± 0.6	0.7	15.1 ± 2.1	0.5
[Zn(atc-Et)_2_] (**1**)	12.1 ± 4.0	7.1 ± 3.7	1.7	102.0 ± 17.6	0.1	3.9 ± 0.0	3.1	52.0 ± 8.6	0.2	9.4 ± 3.4	1.3
[Zn(atc-Ph)_2_] (**2**)	12.1 ± 3.0	3.3 ± 0.0	3.6	325.0 ± 58.4	0.0	5.5 ± 3.1	2.2	14.1 ± 3.8	1.0	34.1 ± 7.0	0.3
Cisplatin	46.2 ± 4.9	60.3 ± 15.1	0.8	85.0 ± 10.1	0.5	143.3 ± 3.3	0.3	75.4 ± 6.0	0.6	56.5 ± 6.8	0.8

* The SI of each complex was calculated as the ratio of the IC_50_ for MRC-5 cells ^1^/IC_50_ for tumor ^2^ cells. Substances with an SI > 3 are considered to be promising. IC_50_: concentration inhibiting cellular proliferation by 50%; SD: standard deviation; SI: selectivity index.

**Table 3:** LC_50_ of zinc complexes **1** and **2** and cisplatin in *Artemia salina* L.

Complexes	LC_50_ (µM) ± SD
[Zn(atc-Et)_2_] (**1**)	962.6 ± 19.2
[Zn(atc-Ph)_2_] (**2**)	1672.7 ± 15.0
Cisplatin	295.1 ± 35.1

**Table 4:** Crystal data and structural refinement for [Zn(atc-Et)_2_] (**1**) and [Zn(atc-Ph)_2_] (**2**).

	1	2
Empirical formula	C_20_H_26_N_8_S_2_Zn	C_28_H_26_N_8_S_2_Zn
Formula weight	507.98	604.06
Temperature (K)	293(2)	296(2)
Wavelength (Å)	0.71073	0.71073
Crystal system	Monoclinic	Monoclinic
Space group	P2_1_	C2/c
Unit cell dimensions	a = 8.8919(5) Å α = 90° b = 14.738(5) Å β = 103.917(5)° c = 9.248(5) Å γ = 90°	a = 13.4421(10) Å α = 90° b = 18.9640(14) Å β = 95.5940(10)° c = 10.9390(8) Å γ = 90°
V (Å^3^)	1,176.4(10)	2775.2(4)
Z	2	4
Density (calculated) (Mg m^−3^)	1.434	1.446
Absorption coefficient (mm^−1^)	1.246	1.069
Crystal size/mm^3^	0.66 × 0.55 × 0.29	0.41 × 0.09 × 0.08
Theta range for data collection	2.36 to 25.12	1.86 to 25.07
Index ranges	−10←h←10, −17←k←17, −11←l←9	−16←h←16, −20←k←22, −12←l←13
Reflections collected	8001	8543
Independent reflections	[R(int) = 0.0161]	[R(int) = 0.0227]
Abs. Corr.	Semi-empirical from equivalents	Semi-empirical from equivalents
Final R indices [I > 2sigma(I)]	R_1_ = 0.0184, wR_2_ = 0.0484	R_1_ = 0.0296, wR_2_ = 0.0668
R indices (all data)	R_1_ = 0.0189, wR_2_ = 0.0485	R_1_ = 0.0368, wR_2_ = 0.0699
Goodness-of-fit on F^2^	S = 1.047	S = 1.037

V: unit cell volume; Z: number of molecules in unit cell; R_1_ = $\sum ||F_{0}|-|F_{c}||/\sum \left|F_{0}\right|$; wR_2_ = ${\left\{\sum \left[w{\left(F_{0}^{2}-F_{c}^{2}\right)}^{2}\right]/\sum \left[w{\left(F_{0}^{2}\right)}^{2}\right]\right\}}^{\frac{1}{2}}$ where *F*_o_ and *F*_c_ are observed and calculated structure factors; I: reflection intensity; GoodF = S = ${\left\{\sum \left[w{\left(F_{0}^{2}-F_{c}^{2}\right)}^{2}\right]/(n-p)\right\}}^{\frac{1}{2}}$, where *n* is the number of reflections and *p* is the total number of parameters refined.

## Supplementary Materials

Supplementary materials can be found at http://www.mdpi.com/1422-0067/17/5/781/s1.
