# Supplementary material for: Novel Zinc(II) Complexes [Zn(atc-Et)2] and [Zn(atc-Ph)2]: In Vitro and in Vivo Antiproliferative Studies
Source: Int J Mol Sci. 2016 May 21;17(5):781. doi: 10.3390/ijms17050781 (PMC4881598; doi:10.3390/ijms17050781)
Supplement: Supplementary file 1 [file ijms-17-00781-s001.pdf]

Supplementary Information: Novel Zinc(II) Complexes [Zn(atc-Et)₂] and [Zn(atc-Ph)₂]: *In Vitro* and *in Vivo* Antiproliferative Studies

Erica de O. Lopes, Carolina G. de Oliveira, Patricia B. da Silva, Carlos E. Eismann, Carlos A. Suárez, Amauri A. Menegário, Clarice Q. F. Leite, Victor M. Deflon and Fernando R. Pavan

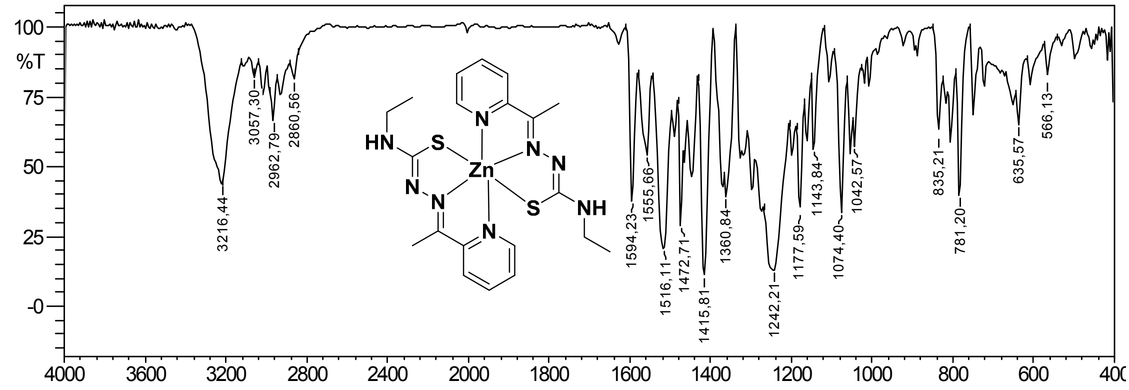

Figure S1. IR spectrum of [Zn(atc-Et)2].

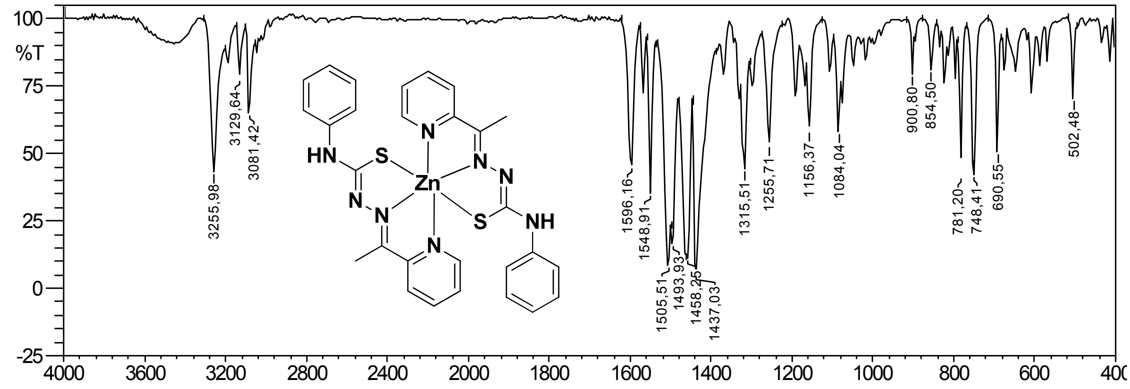

Figure S2. IR spectrum of [Zn(atc-Ph)2].

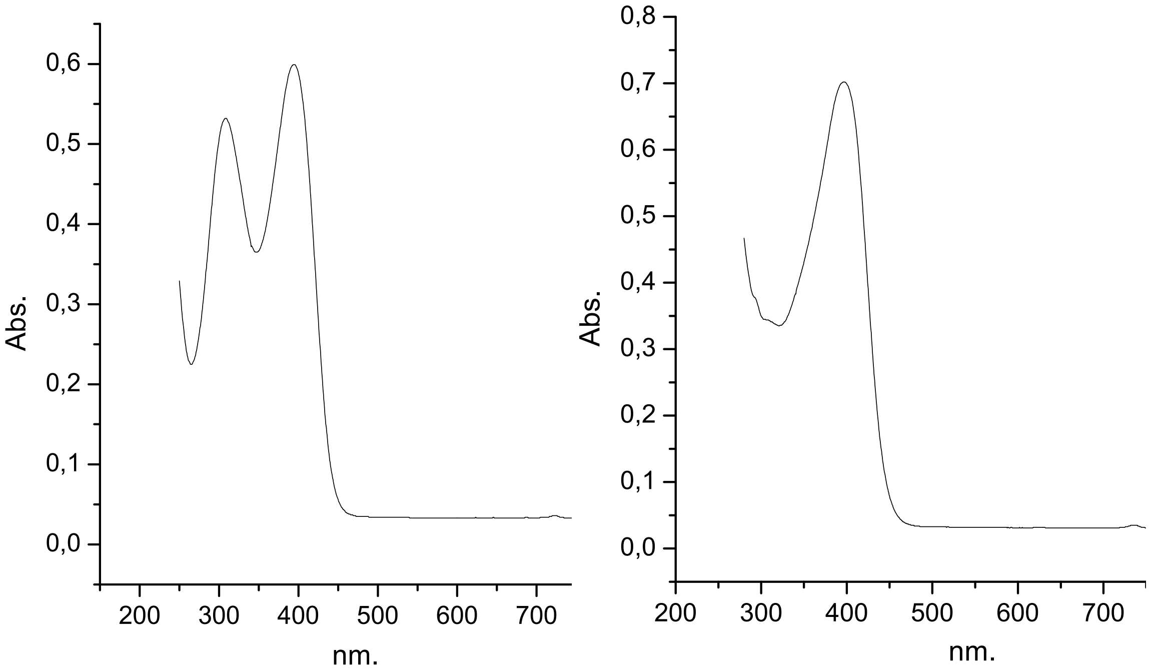

Figure S3. UV visible spectra of complexes [Zn(atc-Et)$_2$] (**left**) and [Zn(atc-Ph)$_2$] (**right**).

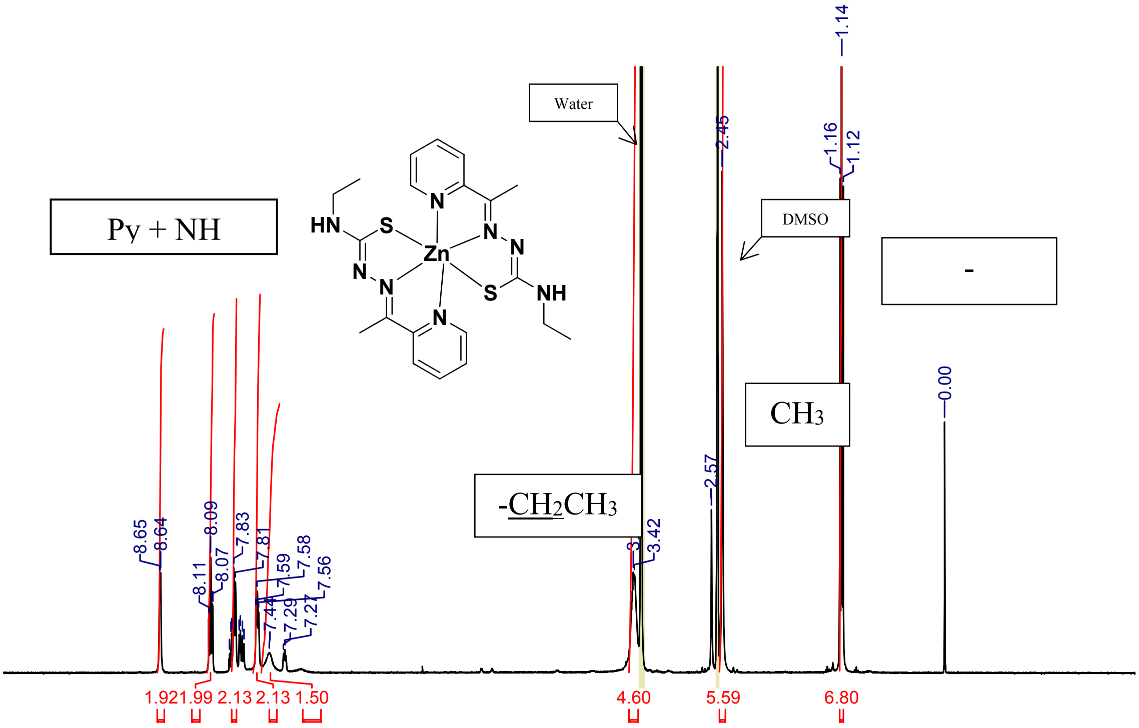

Figure S4. ^{1}H NMR (399.8 MHz, DMSO-d_6) of the complex [Zn(atc-Et)$_2$].

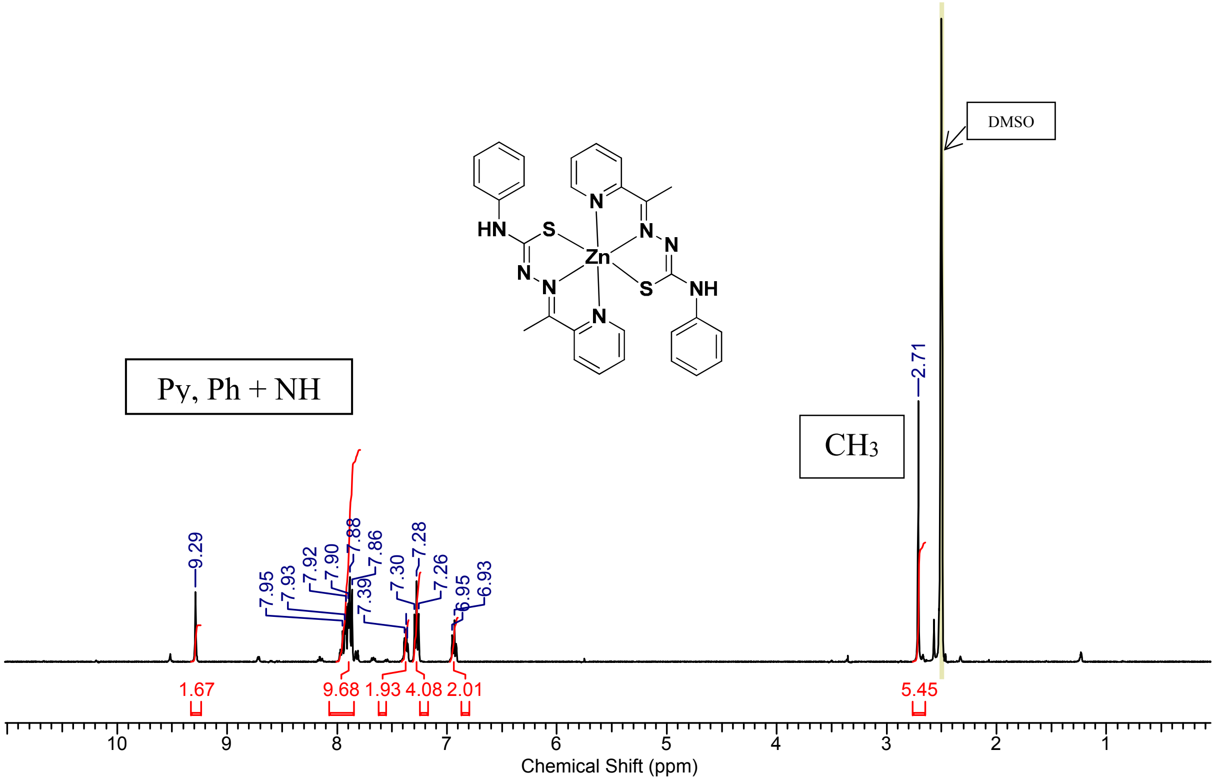

Figure S5. ^{1}H NMR (399.8 MHz, DMSO-d_6) of the complex [Zn(atc-Ph)$_2$].
